# Magnetic resonance imaging-guided and targeted theranostics of colorectal cancer

**DOI:** 10.20892/j.issn.2095-3941.2020.0072

**Published:** 2020-05-15

**Authors:** Yanan Li, Jingqi Xin, Yongbing Sun, Tao Han, Hui Zhang, Feifei An

**Affiliations:** ^1^Department of Radiology, First Hospital of Shanxi Medical University, Taiyuan 030001, China; ^2^Institute of Medical Engineering, Department of Biophysics, School of Basic Medical Science, Health Science Center, Xi’an Jiaotong University, Xi’an 710061, China; ^3^Division of Pharmaceutics, National Pharmaceutical Engineering Center for Solid Preparation in Chinese Herbal Medicine, Jiangxi University of Traditional Chinese Medicine, Nanchang 330006, China; ^4^College of Chemistry and Life Science, Institute of Functional Molecules, Chengdu Normal University, Chengdu 611130, China

**Keywords:** Carcinogenesis, colorectal cancer, magnetic resonance imaging, multimodal diagnosis, targeted theranostics

## Abstract

Colorectal cancer (CRC) is the most common gastrointestinal tract cancer worldwide and is associated with high morbidity and mortality. The development of nanosized drug delivery systems has provided a new direction in CRC treatment. Among these systems, magnetic nanoparticle (MNP)-based multifunctional platforms provide a novel strategy for magnetic resonance imaging (MRI)-related cancer theranostics. At the beginning of this original review, the carcinogenesis and treatment status of CRC are summarized. Then, diversified preparation and functionalization methods of MNPs are systematically analyzed, followed by MRI-involved theranostic strategies. The latest progress in MRI-mediated multimode diagnosis and image-guided targeted therapy in CRC management is the main focus. Finally, the major challenges in promoting MRI-induced precise theranostics of CRC in clinical practice are discussed.

## Introduction

Colorectal cancer (CRC) is the third most commonly diagnosed malignancy (following lung cancer and breast cancer) and the fourth leading cause of oncological mortality worldwide^1^. It represents a significant health issue with over 1.4 million new diagnoses and more than 35%–50% deaths each year since 2012^2^. The incidence and mortality of CRC is correlated with human development and its ranges, predicting that its burden will increase by 60% to more than 2.2 million new cases and 1.1 million deaths by 2030^3^. This status shows that CRC is a significant global problem, which requires intensive study to improve early detection and precise treatments.

Accurate and minimally invasive diagnoses are essential for cancer prevention and the management of affected individuals^4^. Clinically popular diagnostic techniques for CRC include colonoscopy^5^, computed tomography colonography (CT or CTC)^6,7^, magnetic resonance imaging (MRI)^8,9^, positron emission tomography (PET)^10,11^, endorectal ultrasound (ERUS)^12,13^, fluorescence imaging (FI)^14–16^, and genetic detection^17^. Among these techniques, magnetic nanoparticle (MNP)-based MRI has been frequently used in targeted cancer theranostics because of its high magnetic susceptibility, biocompatibility, stability, economy, and diversity of preparation methods^18^.

Surgery represents the mainstay of CRC treatment in early stage cases, which have a more favorable prognosis. Unfortunately, most patients are usually diagnosed in advanced or metastatic disease stages^4^. Neoadjuvant therapy is therefore needed to delay disease progression, prolong survival, and maintain the quality of life. Precise diagnosis derived from MRI and mediated targeted therapy is therefore essential to overcome chemoresistance and accelerate tumor ablation^19^.

In the present review, MRI-mediated targeted diagnosis and therapy for CRC are comprehensively described from the following four perspectives: (1) carcinogenesis, diagnosis and therapy of CRC; (2) MNP-based MRI and theranostic strategies; (3) MRI-mediated targeted theranostics of CRC; and (4) critical challenges concerning successful clinical translation of the multifunctional platforms.

### CRC carcinogenesis, diagnosis, and therapy

CRC is a major public health problem and the leading cause of mortality and morbidity worldwide. Elucidation of pathogenesis is a prerequisite for its early diagnosis, personalized therapy, and reasonable prognosis. In this section, the carcinogenesis of CRC is characterized by identifying the cancer-associated mutations, followed by current diagnoses and treatment approaches using a multidisciplinary model.

#### Carcinogenesis of CRC

CRC is divided into three main types: sporadic, familial, and hereditary CRC. Sporadic CRC arises from somatic mutations and accounts for approximately 70% of all CRCs. Familial CRC makes up 10%–30% of the cases, in which familial predisposition, rather than Mendelian inheritance, is the only disease cause. Genetically, familial CRC is associated with germline minor variants and single-nucleotide polymorphisms (SNPs) of oncogene or tumor suppressor genes. In contrast, inactivating mutations in the same genes cause hereditary CRC, which mainly includes hereditary nonpolyposis colorectal cancer and adenomatous polyposis syndrome, accounting for 5%–7% of all CRCs^20^.

Tumorigenesis of CRC begins with transition of the normal gastrointestinal epithelium to a hyperplasia state, along with increased cell proliferation. Physiologically, a pool of colon stem and progenitor cells is located at the bottom of the crypt. They migrate along the crypt-villus axis and simultaneously differentiate in all epithelial colon lineages, such as Paneth, goblet, enterocyte and enteroendocrine cells, following programmed apoptosis upon arrival at the top of the villus^21^. Under hyperplasia, the epithelium loses its characteristic architecture and organization, becomes dysplastic, and develops into an innocuous adenoma, which is usually a polyp that stems from the hyperproliferative epithelium and protrudes into the colonic lumen. When adenomas encounter the tumoral genetic and immunological microenvironment changes, they will invade into the submucosa and become cancerous. With the aggravation of malignancy, the tumor will spread throughout the body^22^. This process depends on a series of genetic and inflammatory-immunological factors, such as wingless/integrated (Wnt), bone morphogenetic protein (BMP), and transforming growth factor-β (TGF-β), and extracellular matrix and stromal cells, to enable and shape a tumorigenic state following specific mechanisms^23^.

Mechanistically, CRC is a highly heterogeneous disease because of genetic mutations and imperfections. CRCs arise from single or a combination of three different mechanisms, namely, chromosomal instability (CIN), CpG island methylator phenotype (CIMP), and microsatellite instability (MSI)^24^. The CIN pathway begins with mutations in adenomatous polyposis coli (APC), followed by mutational activation of the Kirsten rat sarcoma viral oncogene (KRAS) and inactivation of the tumor suppressor gene tumor protein, p53 (TP53)^25^. The CIMP pathway is important in promoting hypermethylation of various tumor suppressor genes, mainly methylated-DNA-protein-cysteine methyltransferase and DNA mismatch repair protein, together with mutations of the V-raf murine sarcoma viral oncogene homolog B1 (BRAF) and MSI^26^. The MSI pathway is associated with genetic inactivation in short repeated sequences, specifically in DNA mismatch repair genes. The MSI process is often associated with the CIMP pathway, dominating familial Lynch syndrome (LS) and ~15% of the sporadic CRC cases^27^. It symbolizes proximal colon and poor differentiation but has a better prognosis^28^. It is worth stressing that the three mechanisms often coexist and work together in CRC by orchestrating relevant signaling pathways and molecular connections.

The key signaling pathways and mechanisms involved in CRC include but are not limited to Wnt/APC/β-catenin, phosphoinositide 3-kinase (PI3K)/AKT/glycogen synthase kinase-3β (GSK-3β), Ras/Raf, NF-κB, transforming growth factor-β (TGF)-β/Smad, epithelial-to-mesenchymal transition (EMT), and mismatch repair genes. It is assumed that Wnt signaling promotes tumor cell proliferation, inhibits differentiation, and mediates endothelial function by connecting GSK-3β, β-catenin, E-cadherin, APC, and Norrin^29^. The PI3K/AKT/PTEN pathway is often functionally disordered in sporadic and hereditary CRC, activates cell growth, and inhibits apoptosis under extracellular stimulation, such as in the presence of growth factors, cytokines, hormones, heat, oxidative stress, hypoxia, and hypoglycemia^30^. Ras/Raf is a carcinogenic pathway that activates transcriptional regulation and cell cycle progression in response to several types of extracellular stimulations *via* the MEK, ERK, Myc, and cyclin-D/CDK cascades^31^. The NF-κB pathway consists of five subunits that act as transcription factors (RelA/p65, c-Rel, RelB, p50/NF-κB1, and p52/NF-κB2), which participate in cell proliferation and inflammation by dimerization or are sequestered in the cytoplasm by Iκb proteins^32^. GSK-3β is a tumor promoter, and protein crosstalk between Wnt and NF-κB positively regulates NF-κB activity and confers selective growth of CRC cells^33^. The EMT is a common process, in which a group of transcription factors and the tumor microenvironment (TME) are involved through diverse signals, such as Snail, Slug, ZEB1, Twist, TGF-β, EGF, Wnt, and Notch^34^. The reversible EMT is regulated by the TGF-β/Smad pathway and plays a critical role in the early invasion and metastasis of CRC^35^.

These alterations of signal connections confer individual susceptibilities to cancers, and also represent biomarkers for cancer diagnosis and therapeutic intervention.

#### Diagnosis of CRC

Accurate diagnosis is a prerequisite to the selection of a correct treatment strategy and the manipulation of surgical, adjuvant, and palliative therapies^36^. Diagnostic protocols for CRC that have been widely used include colonoscopy, CT or CTC, MRI, PET, ERUS, FI, and genetic detection, among others.

A complete colonoscopy coupled with a histopathological biopsy is considered the gold standard of CRC detection, based on its high diagnostic performance to locate tumors and simultaneously guide the endoscopic excision of polyps^37^. However, the best results are found for lesions > 6 mm, because a complete colonoscopy due to poor bowel condition and tolerance, obstruction, or other technical difficulties, cannot be detected in a substantial number of patients^38^. As a potential alternative to endoscopy, CT or CTC has been shown to be a highly sensitive and specific diagnostic modality for lesions > 10 mm, especially for stenotic tumors, which are difficult to obtain a complete colonoscopy^39^. When lesions are detected by CTC, the next logical step is to use imaging techniques for cancer staging and confirmation, and to return to a colonoscopy for biopsies and immediate polypectomy. The recognized imaging modalities for CRC staging are chest/abdomen/pelvis CT, FI, PET, and MRI. Typically, indocyanine green-based fluorescence imaging has emerged as a potential imaging modality for detecting hepatic, lymph node, and peritoneal metastases of CRC. It has played a vital role in locating metastatic deposits, allowing better staging, orientating complete surgical resection with a prognostic benefit, and reducing the anastomotic leakage (AL) rate after colorectal surgery^40^. PET, as a noninvasive functional imaging method, has unique superiority in providing the spatial information and biological characteristics of tumorigenesis, progression, and metastasis^41^. In recent years, PET alone or in conjunction with CT scanning (PET/CT), possesses excellent sensitivity, specificity, and accuracy, and has played a crucial role in the preclinical and clinical management of CRC, including diagnosis, initial staging, restaging or recurrence, treatment optimization, efficacy monitoring, metastasis evaluation, and prognosis^42^. ERUS is another effective tool for evaluating the integrity of the rectal wall layers, differentiating CRC staging, and detecting regional adenopathy^43^. In contrast, a lower accuracy in identifying nodal involvement has emerged when compared with CT and MRI, and thus the combination of MRI with ERUS provides improved diagnosis^44^.

In short, every modern imaging modality has both strengths and weaknesses, and no technology is considered universally superior. Furthermore, in clinical practice, the selection of diagnostic and therapeutic programs should comply with the international tumor theranostic standards and guidelines such as the National Comprehensive Cancer Network (NCCN)^45,46^ as well as the American Society of Colon and Rectal Surgeons (ASCRS)^47,48^. Typically, both guidelines recommend routine preoperative CT scans of the chest, abdomen, and pelvis for radiographic staging of CRC. They also recommend routine preoperative staging using either ERUS or high resolution pelvic MRI. If the lesion is diagnosed as locally progressive, the patient should be treated with neoadjuvant chemotherapy and radiotherapy^46^. Once the resection has been performed, the chest, abdomen, and pelvis should be scanned by CT annually for the first 5 years^45–47^. At present, there is no specific regulation for routine PET/CT, MRI, or ultrasonography after radical resection, but they can be selectively adopted depending on the disease situation.

Among various diagnostic techniques, MRI is superior for imaging and image-guided drug delivery due to its outstanding magnetism, biocompatibility, biodegradability, targeting modifiability, and the chemical stability of MNP-loaded drug delivery systems (DDS)^49,50^.

#### Therapy for CRC

For very early primary CRC without systemic disease and for malignant polyps, surgery is the usual treatment, involving complete mesocolic excision using a traditional transanal procedure or a video-assisted technique, after adequate staging by ERUS, MRI, and CT^51^. As CRC progresses to the intermediate and locally advanced stages, an adjuvant and neoadjuvant approach is desirable to reduce the local recurrence rate from 30%–40% down to 5%–10% or even lower^52^. Neoadjuvant chemoradiation between long-course chemoradiation and short-course radiation was the standard choice in a German trial^53^.

Chemotherapy coupled with surgery is the usual treatment of metastatic CRC treatment and the only means of enhanced survival. After decades of development, however, the antimetabolite, 5-fluorouracil (5-FU), is the only chemotherapeutic agent used, and represents the foundation of therapeutic regimens from a singular drug to targeted and multiple cytotoxic agents^54^. Trends in treatments for metastatic CRC can be systematically categorized into several eras based on the introduction of new chemotherapeutics and novel combinations. They include 5-FU^55^, leucovorin^56^, capecitabine^57^, cisplatin^58^, oxaliplatin^59^, irinotecan^60^, FOLFOX [folinic acid (leucovorin) + 5-FU + oxaliplatin (elotaxin)]^61^, FOLFIRI [folinic acid (leucovorin) + 5-FU + irinotecan (CPT-11)]^62^, FOLFOXIRI [folinic acid (leucovorin) + 5-FU + oxaliplatin (elotaxin) + irinotecan (CPT-11)]^63^, XELOX [capecitabine (xeloda) + oxaliplatin (elotaxin)]^64^, and XELIRI [capecitabine (xeloda) + irinotecan (CPT-11)]^65^. Importantly, with the incorporation of platinum agents, plant alkaloids, and antibiotics in adjuvant and neoadjuvant chemotherapy, combination prescription clearly enhanced the 5 and 10 year survival rates for CRC patients.

Although progress has been made in the chemotherapeutic treatment of CRC, clinical application of chemotherapeutics is hindered because of toxicity and a lack of specificity. Targeting the specific elements of cancer signaling pathways will provide a new solution and generate an innovative option to improve therapeutic efficacy and specificity. Previous studies have shown that receptor tyrosine kinases (RTKs) play an essential role in processing tumor growth and progression by regulating cell proliferation, apoptosis, and angiogenesis^66^. This important role of RTK, the action site of molecular targeted agents, may be divided into three categories according to the drug-signal mechanism: (1) monoclonal antibodies against vascular endothelial growth factor (VEGF) and epidermal growth factor receptor (EGFR); (2) recombinant fusion proteins against angiogenesis; and (3) molecules that inhibit RTK.

Due to the overexpression of EGF and VEGF in CRC, considerable research has been undertaken to develop inhibitory treatments against them for anticancer treatments. Considering the most typical examples corresponding to the above classification, bevacizumab is a humanized monoclonal antibody that binds to soluble VEGF, prevents receptor binding, and inhibits endothelial cell proliferation and vessel formation. Cetuximab is a recombinant human/mouse chimeric monoclonal antibody that binds to the extracellular domain of human EGFR, competitively inhibits the binding of EGF and other ligands to EGFR, blocks receptor phosphorylation and activation of receptor-associated kinases, and consequently inhibits the signaling cascade^67^. Panitumumab is a recombinant human monoclonal antibody that binds to EGFR, blocks the binding of EGFR ligands to cancer cells, and inhibits EGF dependent tumor cell activation^68^. Aflibercept is a recombinant fusion protein that contains VEGF-binding portions fused to human immunoglobulin IgG1, and acts as a high affinity ligand trap, to block the activity of VEGF primarily involved in angiogenesis, vasculogenesis, vascular permeability, and placental growth factor by preventing ligand binding to their endogenous receptors^69^. Regorafenib is an oral multikinase inhibitor that targets angiogenic, and stromal and oncogenic RTKs^70^. It is the first small molecule multikinase inhibitor to improve survival in metastatic CRC that has been refractory to all standard therapies, but it only provides benefit to patients with a good physical condition and organ function, due to its serious side effects including specific hand-foot-skin reactions, fatigue, and elevated liver enzymes.

In addition, an increasing number of molecular targeted drugs and miscellaneous therapeutic schedules have been reviewed in a previous report, providing guidance for synergistic therapy and imaging-mediated CRC management.

### MNP-based diagnostic and therapeutic potentials

#### Preparation and functionalization of magnetic nanoparticles (MNPs)

MNPs have generated great interest in the field of cancer nanotheranostics because of their intrinsic physical properties, magnetic susceptibility, biocompatibility, stability, and other related positive characteristics. MNPs are usually composed of three components: a magnetic core, polymer coating, and functional moieties. Their magnetic property enables their use as contrast agents in MRI and as a therapeutic system in hyperthermia. Their physicochemical features and biocompatibility endow them with the potential to deliver bioactive cargos and specific ligands for targeted therapeutic regimes.

The magnetic properties of MNPs are strongly related to their composition, morphology, size distribution, and surface chemistry, which depend on the selection of preparative methods. MNPs can be synthesized *via* mechanical attrition (top-down) or chemical processes (bottom-up). Compared with the physical approach, chemical methods are more effective for the synthesis and property control of monodispersed MNPs. Chemical protocols include coprecipitation^71^, thermal decomposition^72^, hydrothermal synthesis^73^, microemulsification^74,75^, sol-gel reactions^76,77^, polyol synthesis^78^, and electrochemical^79^ and sonolysis^80^ methods. The implementation process and advantages and disadvantages of each method are summarized in **Table 1**.

The biodegradable Fe core of MNPs can be synthesized to be superparamagnetic. A polymer coating is generally placed around the Fe core, to protect it from agglomeration and oxidation and to serve as a template for targeting agents, imaging tags, and delivering therapeutic payloads. Surface modification has become an integral part of the MNP design. These can modify the functionalities through different techniques including functionalization, coating, and encapsulation. Several commonly used polymer-based MNP encapsulation techniques include nanoprecipitation from preformed polymers such as polyethylene glycol (PEG) and its derivatives^81^, simple emulsion evaporation (SEE)^82^, double emulsion evaporation (DEE)^83^, and layer-by-layer (LBL) assembly^84^. They are detailed in **Table 2**.

#### Diagnostic potency of MNPs

The excellent magnetism of MNPs permits their detection by MRI, which is one of the most powerful noninvasive techniques currently applied clinically. The MRI principle is based on the hydrogen protons present in the body, which become excited and align themselves along the magnetic field when they interact with a transverse radiofrequency pulse and are subsequently relaxed and return to their original state. During the process, the local variation in proton density of the tissue results in the production of highly detailed MRI images. As contrast agents, MNPs that have accumulated in the tissue provide MR contrast enhancement by shortening both the longitudinal (T1) and transverse (T2) relaxation of the surrounding protons.

The size, composition, surface properties, and polymer stabilizers of MNPs can influence the relaxation of water molecules and resulting MRI efficiency. In this regard, although several Fe_3_O_4_ NP contrast agents, such as Abdoscan^®^, GastroMARK^®^, Lumiren^®^, Resovist^®^, Cliavist™, Feridex^®^ and Endorem™ have been commercialized to improve cancer detection, diagnosis, and therapeutic management of solid tumors, an increasing number of studies have focused on the surface modifications of MNPs to enhance their biocompatibility and targeting^85^.

The imaging capability of MNPs by MRI has also prompted the development of MR-based biosensors. In the presence of specific enzymes or chemical compounds, the reversible assembly and disassembly of sensors results in a change in transverse magnetic relaxivity. This discovery stimulated the emergence of numerous biosensors to detect proteases, oligonucleotides, enantiomeric impurities, and active pharmaceutical ingredients.

#### The imaging-synergized therapeutic potency of MNPs

Theranostics has emerged as an efficient strategy for precision or personalized medicine. It involves “NP-based drugs” and improves the diagnosis and treatment efficacies of cancers with limited systemic toxicity, while the therapeutic outcome can be rapidly reviewed to plan subsequent options. The unique physicochemical properties of MNPs endow them with great promise for building various theranostic systems to facilitate MRI detection and accurate treatments, thus providing a significant advance in personalized therapy.

In clinical practice, a single molecular diagnostic modality cannot provide complete structural and functional information concerning the object simultaneously, and consequently, multimodal diagnosis combining two or more imaging techniques has emerged to accurately assess tumor information. Coincidentally, MNPs represent a powerful multifunctional probe for MRI-based multiple imaging applications by integration with other diagnostic schemes, such as FI^86^, upconversion luminescence (UCL)^87^, CT, PET^88^, focused ultrasound^89^, photoacoustic (PA) imaging, and Raman imaging^90^.

Nanotheranostics is the combination of diagnosis and therapy in a single nanoplatform. It emphasizes the MRI-mediated versatile nanoformulations by combining with chemotherapy^91^, phototherapy^92,93^, targeted therapy^94^, gene therapy, chelation therapy^95^, or magnetic hyperthermia (MHT).

Another significant application of MNPs is to exploit their therapeutic response *via* hyperthermia. To induce MHT, the tissue is exposed to an abnormally high temperature (41–47 °C) generated by hysteresis loss when MNPs are exposed to a magnetic field with an alternating current. Cancer cells can be damaged and killed by heat for remedial purposes. In comparison to surgery, radiotherapy (RT), and chemotherapy, and MHT provide a new therapeutic approach based on the following advantages: (1) the nanosize effect of MNPs guarantees their capacity for passive targeting and minimally invasive injection; (2) the stimulus responsiveness and active targeting can be modulated by chemical modification of MNPs with specific elements in the preparation process; (3) the MRI function is acquired simultaneously with MHT; and (4) MHT has negligible systemic toxicity, selectively attacking tumor cells and evading normal cells under heat diffusion^96^.

Driven by the aforementioned principle, hyperthermia-derived applications of MNPs utilize three approaches: MHT, MRI-cooperated MHT, and magnetothermal-responsive drug release from MNPs. In hyperthermia-based DDS, thermoresponsive polymers are introduced to modify the surface of MNPs, and they simultaneously act as carriers for drug loading *via* covalent conjugation or physical adsorption. When exposed to magnetic heat, the thermosensitive linkers are broken, enhancing the permeability of polymeric carriers, and releasing their cargos *via* a bond-breaking mechanism. Recently, Fe^3+^-containing hardystonite (Fe-HT) scaffolds have been prepared, which possess highly specific surface areas for the regeneration of large bone defects caused by malignant bone tumors, through a combination of hyperthermia, local drug delivery and osteoconductivity^97^. Thermo-responsive magnetic ammonium bicarbonate (MagABC) liposomes are designed to conduct magnetic targeting and thermoresponsive-controlled release of doxorubicin (DOX) for hyperthermia-triggered local drug delivery^98^. Furthermore, Zamora-Mora et al.^99^ crosslinked chitosan NPs ionically with tripolyphosphate salts, to obtain core-shell multipurpose nanocarriers for combined 5-FU delivery and improved MHT.

### MRI-guided theranostic applications in CRC

The potential use of nanotechnology in cancer theranostics has stimulated great interest among researchers in recent years. In particular, MNPs have emerged as a promising platform for MRI-based multimode imaging, theranostics, and efficacy monitoring of malignant tumor treatments. Among numerous achievements, the latest representative progress in MNP-based theranostic applications in CRC are discussed below.

#### MRI-based multimode imaging in CRC

In a previous report, several commonly used imaging techniques, such as colonography, CT, CTC, MRI, and PET/CT, were systematically introduced^100,101^. Soon thereafter, Saing et al.^102^ developed an analytical model to balance the diagnostic accuracy, sensitivity, specificity, and cost-effectiveness between MRI and CT in patients with liver metastatic CRC. The clinical results supported the superior sensitivity (0.943 *vs.* 0.768) and cost advantage of MRI *vs*. CT, and MRI/CT was recommended as a complementary technical combination to improve the diagnostic accuracy for earlier, curative, and disease management of metastatic CRC^102^. The ability of MRI to discriminate local staging of CRC was then integrated with PET for higher diagnostic performance, liver metastasis evaluation, as well as to provide additional information about the disease phenotype and the biology of CRC. Whole body PET/MRI will provide a technical option to improve the clinical diagnosis of both primary and metastatic CRCs^103^. Similar results were verified by Brendle et al.^104^ in a successive pilot study of metastatic CRC.

CTC with multiplanar reconstruction (MPR) and MRI has been used to evaluate the accuracy of preoperative T staging of 45 consecutive patients with very low rectal adenocarcinoma. Higher accuracy (89% *vs.* 71%) and better sensitivity (82% *vs.* 53%) were acquired from CTC with MPR than with MRI, indicating more precise preoperative T staging of lower CRCs for selecting intersphincteric resections, abdominoperineal resections (APRs), and neoadjuvant therapy^105^. However, MR colonography (MRC) was introduced as a minimally invasively screening tool to detect CRC and its precursors, reduce morbidity and mortality, and improve patient acceptance and participation^106^. In a clinical study including 99 CRC patients, no patients experienced severe or extreme MRC bowel preparation burden compared with 31.5% of the patients with colonoscopy bowel preparations. Subsequently, lower overall burden was encountered in the process of MRC (5.2%, limited carbon dioxide insufflation) than conventional colonoscopy (25.6%, *P* < 0.0001), and a larger proportion of patients (61.4%) preferred MRC to colonoscopy (29.5%) immediately after the examinations and 5 weeks later (57.0% *vs.* 39.5%)^107,108^. More desirable diagnostic value was also achieved with MRC when compared with CTC *via* preferred reporting items for systematic reviews and meta-analyses (PRISMA) of 23 studies on the diagnosis of CRC, focused on the sensitivity, specificity, positive likelihood ratio, negative likelihood ratio, and receiver operating characteristic curve^109^. Ileva et al.^110^ also developed a reliable noninvasive method, called MRI virtual colonography, to monitor CRC and mucosal inflammation in a mouse model. After intravenous injection of gadolinium-based contrast agent, the inflamed colonic wall, tumor vascularity, and developmental stage of CRC were clearly distinguished and visualized for monitoring the prevention and treatment of tumors within 1.5 h^110^. A novel MRC technique was developed by designing multifunctional solid lipid NP-loaded gadolinium diethylenetriamine pentaacetic acid and octadecylamine fluorescein isothiocyanate simultaneously as a tumor-absorbable NP contrast agent, so that both human (HT-29) and mouse (CT-26) colon carcinoma could be enhanced and evaluated in a mouse model *via* direct absorption or uptake of the NP contrast agent^111^.

In brief, MRI has been developed as a valuable tool and is the core of multifunctional theranostics for preoperative evaluation, multidimensional diagnosis, and image-guided therapy of patients with CRC.

#### MRI-guided targeted theranostics in CRC

Nanotheranostics that incorporate therapeutic agents, targeting groups, and diagnostic probes are emerging as the next generation of precision components to improve the localization of lesions and the therapeutic outcome of cancer. To achieve this goal, MNP-based MRI technology has been incorporated within multifunctional systems. The latest developments in MRI-guided targeted theranostics in CRC by integrating MRI with chemotherapy, photothermal therapy (PTT), photodynamic therapy (PDT), gene therapy, and combination therapy are discussed and are summarized in **Table 3**^112–130^.

#### MRI-guided chemotherapy in CRC

The main drawbacks of chemotherapy involve nonspecific action, rapid clearance from the system, and side effects affecting healthy tissues. To overcome these shortcomings, MNPs have served as a support for drug loading by covalently attaching or mechanically encapsulating therapeutic agents onto or within a polymer-magnetic nanocomposite. The nanosize effect, magnetic targeting, and site-specific delivery of cargos protect the active pharmaceutical until it reaches the target cancer cells.

In 1976, Zimmermann and Pilwat used magnetic erythrocytes to deliver cytotoxic drugs, thus facilitating chemotherapeutics carried by MNPs^131^. The first application of MNP-drug (epirubicin) in an animal model was reported by Lubbe et al.^132^, who targeted pancreatic cancer. In addition, using a porcine model of hepatocellular carcinoma, Goodwin et al.^133^ achieved external magnetic field-driven accumulation of DOX-loaded MNPs at the tumor location without any off-target toxicity. Since then, a variety of MRI-guided chemotherapeutic approaches have been used in targeted the theranostics of CRC.

A strategic report was focused on recent advances and the emerging possibility of MNP technology in enhanced therapeutic applications against CRC. Subsequently, pH-sensitive triblock copolymer-based nanomicelles (PEALCa) were synthesized from PEG, poly[N-(N′,N′-diisopropylaminoethyl) aspartamide] [P(Asp-DIP)], and poly (lysine-cholic acid) [P(Lys-Ca)]. These micelles self-assembled into stable vesicles of ~50–60 nm in a neutral environment to encapsulate the anticancer drug paclitaxel (PTX) and hydrophilic superparamagnetic iron oxide (SPIO), to avoid uptake by the reticuloendothelial system (RES). *In vitro* studies had shown that after internalization by a human CRC cell line (LoVo cells), PTX-SPIO-PEALCa micelles became lodged inside lysosomal compartments (pH ~ 5.0) and rapidly released PTX. The drug delivery effect was verified by MRI and histology analyses in a CRC xenograft model (LoVo cells). Moreover, effective suppression of CRC tumor growth was achieved by the PTX-SPIO-PEALCa group, with an average tumor volume of 65.0 ± 8.4 mm^3^ at day 30, which indicated a negligible tumor growth when compared to the generic Taxol^®^ group of 598.7 ± 77.4 mm^3^ and the phosphate-buffered saline (PBS) group of 1050.7 ± 54.4 mm^3^ (*P* < 0.001). These results supported PTX-SPION-PEALCa pH-sensitive micelles as a promising MRI-visible DDS for CRC therapy^112^.

In another study, Boissenot et al.^113^ encapsulated a core of perfluorooctyl bromide (PFOB) into a shell of poly(lactide-co-glycolide)-PEG with PTX loading by a modified emulsion-evaporation method into nanocapsules of 120 nm in diameter. The nanocapsules appeared similar at an *in vitro* half-maximal inhibitory concentration (IC_50_) with a generic Taxol^®^ formulation (5 × 10^−5^ mg/mL) in CT-26 CRC cells. However, in CT-26 xenograft tumors, ^19^F-MRI showed that the PTX-loaded nanocapsules passively accumulated at tumor sites and led to a promising and statistically significant two-fold reduction of tumor growth compared to the negative control and Taxol^®^ group^113^. In a similar manner, 5-FU-loaded poly(lactic-co-glycolic acid) magnetic nanocapsules were synthesized with a diameter of ~67.2 nm. A sustained *in vivo* release profile and prolonged lifetime were found in rabbit plasma. MRI revealed the increased tissue affinity and tumor enrichment of nanocapsules, which resulted in more efficient tumor volume inhibition (100 mm^3^ at day 21) than 5-FU alone (1,500 mm^3^, *P* < 0.01)^114^. These results supported drug-loaded nanocapsules as a potential theranostic platform for effective CRC inhibition.

Voulgari et al.^115^ prepared core-shell magnetic nano-assemblies by hydrolytic alkaline precipitation of a single iron molecular precursor in the presence of poly[(methacrylic acid)-g-poly(ethylene glycol methacrylate) P(MAA-g-EGMA)] as an *in situ* coating agent. By encapsulation of cisplatin as a cytotoxic drug, the magnetic DDS displayed excellent MRI and anticancer properties in a cisplatin-resistant HT-29 human colon adenocarcinoma cell culture model. The tumor volume of the magnetic DDS group at the end of the study period (day 38) was 400 mm^3^, which was more effective than free cisplatin (900 mm^3^), and was particularly enhanced by the external magnetic field (300 mm^3^)^115^. To further prevent the biotoxicity of platinum drugs, PEGylated multiwalled carbon nanotubes (MWNTs) decorated with SPIO were developed to encapsulate oxaliplatin for sustained drug release and MRI analyses, with only 36.25% of oxaliplatin leakage within 12 h and 55.48% over 144 h. The optimal cytotoxicity was observed at 96 h due to the internalization peak of oxaliplatin in HCT-116 human CRC cells. In HCT-116 tumor-bearing mice, Oxa/MagMWNT-PEG showed more effective tumor inhibition compared with the Oxalip group (10 mg/kg), with only trivial weight loss (6.25%) and organ toxicity. Importantly, the tumor boundary could be enhanced by MRI to manage the therapeutic effect and treatment option^116^.

For structural innovation, a SPIO nanorod (SIONR) core was conjugated to a pluronic F127 shell, which was prepared using a hydrothermal method. After loading PTX through hydrophobic interactions, the obtained PTX-F127-SIONR nanocarriers elicited a more effective concentration-dependent inhibition of CT-26 cell proliferation than PTX, with a cell viability after 24 h of incubation of 23.2% and > 58.6% (*P* < 0.05), respectively. In CT-26 xenograft tumor mice, the PTX-F127-SIONR group showed a higher therapeutic response and survival rate with reduced tumor growth than the PTX group, resulting in a shorter survival time and comparable tumor size as the control group, and 100% death at day 34 after injection. Moreover, the lesion localization and state change of small tumors could also be visualized by enhanced MRI^117^.

In addition, the drug loading method and magnetic material type were also important factors determining the drug cytotoxicity and imaging results. Augustin et al.^118^ adopted a promising strategy to conjugate Fe_3_O_4_ MNPs to DOX to investigate the cytotoxic effects and cell death processes in HT-29 cells. DOX-NPs showed higher cytotoxicity (IC_50_ = 0.245 μmol/L) than free DOX (IC_50_ = 0.757 μmol/L), which resulted in G2/M arrest followed by late apoptosis and necrosis at the IC_50_ concentration^118^. Other kinds of contrast agents, such as Mn^2+^, were selected as examples to explore their theranostic applications in CRC. Meng et al.^119^ initially deposited manganese dioxide on the surface of albumin-bound PTX NPs (ANPs-PTX) to obtain MnO_2_-functioned ANPs-PTX (MANPs-PTX) with a diameter of 140 nm. MANPs-PTX exhibited improved chemoradiation therapy in the CT-26 cell line, with MANPs-PTX+RT, chemotherapy alone (ANPs-PTX), and RT alone cell viabilities of 23.4%, 44.9%, and 52.5%, respectively. In mice bearing CT-26 tumors, MANPs-PTX could consume excess hydrogen peroxide (H_2_O_2_) to improve chemoradiation therapy, exhibiting a maximal tumor growth inhibition of 96.57%, compared with the other treatments with insufficient efficacy. Moreover, the released Mn^2+^ from MANPs-PTX showed excellent T1 MRI performance for tumor detection and treatment monitoring. Taken together with the maintenance of body weights, MANPs-PTX appeared to be a potential theranostic agent with clinical prospects^119^.

#### MRI-guided PTT in CRC

Image-guided photothermal ablation (PTA) has been one of the principal tools in the management of primary CRC or colorectal liver metastases (CRLM). At present, PTT represents a widely used therapeutic option with a superior capacity to treat cancer rather than damage normal tissues. In this model, photothermal agents absorb near-infrared (NIR) light to generate localized hyperthermia through nonradiative transition at the tumor site under laser irradiation. A desired PTT agent should exhibit strong absorbance in the NIR region and low fluorescence emission, typically similar to nanoarchitectures of carbon (C), gold (Au), tungsten (W), copper (Cu), and molybdenum (Mo)-based compounds^134^. However, current PTA techniques mainly apply to tumors less than 4–5 cm in diameter and result in an indistinct tumor boundary and incomplete ablation^135^. As a consequence, MNPs can be integrated into the MRI-PTT system to observe the tumor outline and therapeutic process.

The strong localized surface plasmon resonance absorption of Au nanostructures in the NIR region make them mainstream candidates for PTT^136^. Directed by a systematic overview examining the scientific and practical significance of Au-loaded nanomaterials in the enhancement of PTT^137^, various MNPs-Au nanohybrids were constructed to achieve MRI-guided PTT in CRC. For example, White et al.^120^ prepared monoclonal antibody (anti-MG1)-conjugated magnetic Au hybrid NPs and determined their temperature variation and therapeutic efficacy following irradiation with an 808 nm diode laser in 19 Wistar rats. The mean temperature ± standard deviation in the anti-MG1-coated HNPs, HNPs, and control groups were 50.2 ± 7.8 °C, 51 ± 4.4 °C, and 39.5 ± 2.0 °C, respectively. The corresponding tumor inhibition rates were 38% ± 29%, 14% ± 17%, and 7% ± 8%, respectively (*P* = 0.043). These findings indicated that monoclonal antibody-targeted HNPs provided an effective catalyst for PTA of CRC by increasing the ablation zone^120^.

Aptamer-targeted theranostic agents were engineered by microemulsion preparation of SPIONs, coated with Au NPs and then conjugated to thiol-modified oligonucleotide MUC-1 aptamers. The obtained aptamer-Au@SPIONs (~19 nm) produced significant contrast enhancement in HT-29 cells and an 80% cell inhibition rate at a system concentration of 500 μg/mL and exposure of 820 nm NIR^121^. Another immuno-targeted Au-Fe_3_O_4_ hybrid NP, HNPs-scFv (single-chain variable fragment), was developed to perform MRI-based laser-assisted therapy in a human CRC cell SW1222-bearing xenograft model. Under 808 nm NIR irradiation, > 65% SW1222 cell death suggested effective PTT. The theranostic effectiveness was verified by high contrast T2 MRI and tumor suppression in SW1222 xenografts, manifesting as a difference in tumor volume. The HNPs-scFv treatment group displayed the smallest tumor size of 72 ± 7 mm^3^ compared with the control group (untreated: 193 ± 18 mm^3^), laser only group (161 ± 15 mm^3^), and nontargeted HNPs plus laser group (195 ± 10 mm^3^)^122^. Upon introducing A33 antigen-targeted scFv, the A33scFv-HNPs exhibited an excellent magnetization value of 44 emu/g for MRI potential and a strong optical absorbance at 800 nm. After a 6 min treatment with an 808 nm laser, > 53% of the A33-expressing cells (SW1222) were involved in apoptosis-related cell death compared with < 5% in A33-nonexpressing cells (HT-29). These results supported bioconjugated HNPs as an effective MRI-based antigen-targeted PTT agent^123^.

**Figure 1:**
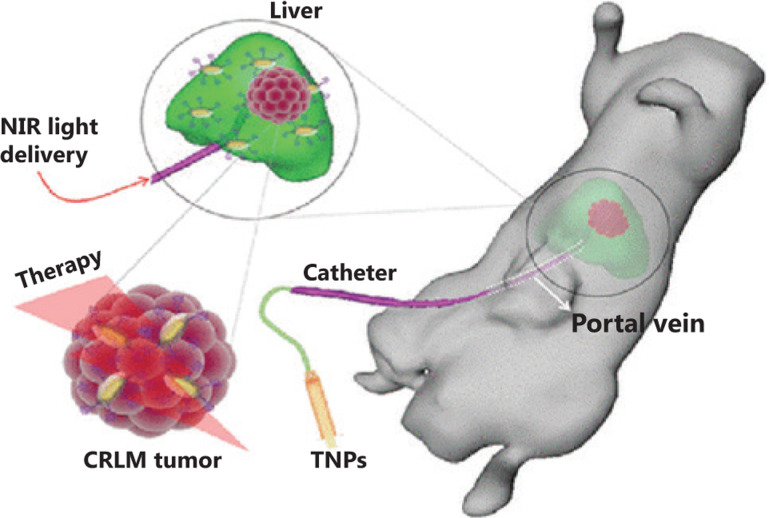
The site-selective delivery of TNPs via the hepatic portal vein and photothermal therapy under a catheter-based 808 nm near infrared laser. Reprinted with permission from Ref. 124, Parchur AK, Sharma G, Jagtap JM, Gogineni VR, LaViolette PS, Flister MJ, et al. Vascular interventional radiology-guided photothermal therapy of colorectal cancer liver metastasis with theranostic gold nanorods. ACS Nano. 2018; 12: 6597-611. Copyright@ American Chemical Society.

By using multiple diagnostic modes, an interventional image-guided PTT agent, sub-100 nm of Au@Gd_2_O_3_: Ln [Ln (lanthanide) = Yb/Er (ytterbium/erbium)], was used for optical/MR/X-ray imaging. The site-selective hepatic image doubled the tumor-to-liver contrast of CC-531 rat CRLM. Under 808 nm NIR light irradiation for 5 min, the temperature of the tumor site increased by ~7.5 °C in the saline group, whereas it increased by ~19.5 °C in the NP group, indicating a stronger PTT effect (**Figure 1**)^124^. Based on this approach, Zhang et al.^125^ developed hybrid anisotropic nanoparticles (HANs) for integration in MRI and photoacoustic imaging (PAI)-directed chemotherapy and thermotherapy. At 24 h after injection of HANs into HT-29-bearing tumor mice, strong MRI and PA signals tracked the distribution of HANs in the whole tumor, guaranteeing the maximum therapeutic effect. Following irradiation with an 808 nm NIR laser, the tumor temperature in the HANs group increased by 25 °C, while it increased only 4 °C in the PBS group. During the drug treatment period, the tumor volume in the HANs + laser group remained ~0 without relapse, with a slightly reduced tumor size in the HANs alone group, while it grew rapidly in the laser without HANs and PBS groups^125^.

The unique structure and NIR absorption properties of graphene make it a very effective option as a PTT component, particularly in MRI-directed CRC theranostics^138^. As an example, Lu et al.^126^ prepared magnetic graphene oxide (MGO) by depositing Fe_3_O_4_ NPs on GO using chemical co-precipitation, modified with PEG and cetuximab (CET, an EGFR monoclonal antibody). The resulting MGO-PEG-CET had a high drug-loading capacity of 6.35 mg/mg for DOX and pH-dependent release behavior. *In vitro*, MGO-PEG-CET/DOX showed a lower IC_50_ toward CT-26 cells at 1.48 μg/mL compared with MGO-PEG/DOX (2.64 μg/mL), and an NIR laser exposure-enhanced cytotoxicity of MGO-PEG-CET/DOX of 1.17 μg/mL due to the combination with PTT. In CT-26 BALB/c mice, the relative tumor volumes in the control (PBS), DOX, MGO-PEG-CET/DOX, MGO-PEG-CET/DOX + magnet, and MGO-PEG-CET/DOX + magnet + laser groups were 12.1, 10.1, 9.5, 5.8, and 0.42, respectively. The prominent MRI and therapeutic efficacy promoted the dual-targeted MGO-PEG-CET/DOX to the forefront of image-combined chemotherapy and PTT of CRC^126^.

Moreover, ultrasmall (5.3 nm) and electrically neutral coordination polymer nanodots (Fe-CPNDs) were scaled-up for pH-activated MRI at the minimum dose of 0.8 mg/kg. Furthermore, the strong absorption of Fe-CPNDs in the visible to NIR region endowed them with PTT potential. Upon 808 nm NIR laser irradiation, the Fe-CPNDs were highly cytotoxic to SW620 human CRC cells in a dose-dependent manner, whereas they showed excellent biocompatibility (> 90% cell viability) with both normal (HL-7702) and tumor cells (SW620), even up to a Fe concentration of 200 μg/mL without illumination. The MRI-guided PTT efficacy was manifested as complete tumor ablation in SW620 mice on day 20. These findings revealed a new class of renal-clearable nanomedicines for MRI-triggered PTT in CRC^127^.

#### MRI-guided PDT in CRC

PDT is an alternative treatment for nonmalignant tumors. During the course of PDT, the photosensitizer (PS) is excited by light with lower optical power densities than the PTT process, which generates cytotoxic reactive oxygen species and causes irreversible cell apoptosis and tissue destruction in a moderate manner, but with minimal toxicity to normal tissues^139^. The vast majority of efficient PSs are hydrophobic, allowing for the introduction of nanocarriers and emphasizing use of MNPs for the development of MRI-based PDT therapeutic platforms for cancer treatment^140^. Among these, a typical case is used to explain the application of the MRI-PDT system in CRC treatment.

In a recent study, Mühleisen et al.^128^ loaded hypericin on SPIONs and guided them to the desired location using an external magnetic field. The toxicity and hypericin-mediated effects on HT-29 cells were characterized and this confirmed that cell proliferation was significantly reduced and even completely abolished at a high hypericin concentration (2 μmol/L) over a long illumination time (60 h). Driven by the double targeting strategy involving magnetic and laser-induced accumulation and photoactivation, the therapeutic efficiency and specificity were improved, thus attenuating toxic side effects in clinical practice^128^.

#### MRI-guided gene therapy in CRC

During the course of effective gene therapy, DNA or RNA is transferred into targeted cells *via* transfection methods for disease therapy. To avoid enzymatic degradation of DNA and RNA and their poor diffusion across cell membranes, magnetofection is used to direct nucleic acid-bound MNPs to target cells under an external magnetic field. Generally, the MNP-containing gene delivery systems are functionalized with a positively charged polymer or targeting group to promote drug release and lesion imaging^141^.

Huang et al.^129^ applied magnetic Au NPs as a nonviral gene carrier to mediate plasmid silencing of Bcl-2-associated athanogene 1 (Bag-1), an anti-apoptotic gene that is highly expressed in CRC, for magnetofection-induced cancer treatment. *In vitro* tests using human CRC LoVo cells revealed cell apoptotic rates in the nanocarrier, plasmid, and nanoplasmid groups of 14.65 ± 0.018, 19.56 ± 0.050, and 47.55 ± 0.022, respectively, and apoptotic/necrotic ratios of 1.016 ± 0.132, 1.415 ± 0.315, and 3.573 ± 0.369, respectively. In LoVo-bearing nude mice, the Bag-1 protein level was silenced to 60% of the control group, which caused a debasement of Wnt pathway molecules, such as C-myc and β-catenin. These findings confirmed the important role of magnetic Au NPs in delivering siRNA plasmid silencing of Bag-1 for magnetism-guided gene therapy of CRC^129^.

Importantly, a multifunctional theranostic micellar DDS was constructed utilizing cationic poly[2-(dimethylamino)ethyl methacrylate]-block-poly(ɛ-caprolactone) to load with SN-38 (7-ethyl-10-hydroxycamptothecin), ultrasmall SPIO (USPIO), and PEG-conjugated VEGF small interfering RNA (siRNA). The SN-38/USPIO/siRNA nanoparticles could target to tumor sites of LS174T-bearing nude mice and significantly inhibit tumor growth. Additionally, the nanoparticles acted as a T2-weighted MRI contrast agent for diagnosis and tracking the therapeutic outcomes^130^.

#### MRI-guided combined therapy in CRC

The multimodality theranostic system involving more than one therapeutic modality has been integrated with multimodal imaging agents to produce a nanoentity, which has shown very promising prospects for cancer treatment. To meet this requirement, MNPs show an ideal capacity as labels for MRI-directed therapeutic synergy. In this section, only representative theranostic schemes are mentioned without detailed and extensive descriptions.

In a previous report, the core-shell-shell NaYbF_4_:Tm@CaF_2_@NaDyF_4_ (Yb: ytterbium, Tm: thulium, Dy: dysprosium, CaF_2_: calcium fluoride) nanocomposite was designed for MRI-enhanced upconversion/CT lymphatic imaging^87^. Novel HANs were then initially utilized for MRI and PAI-induced chemotherapy and thermotherapy^125^. Another research hotspot based on MNPs has been MRI-oriented hyperthermia combined with other therapies, which mainly consist of chemotherapeutics such as DOX, 5-FU, cisplatin, methotrexate, and others^142,143^. In addition, other multipurpose systems, including but not limited to MRI-integrated multidrug chemotherapy, chemo-gene therapy, and stem cell therapy, are constantly emerging for CRC management^144^.

#### Clinical applications of MRI-guided theranostics in CRC

The clinical application of MRI is presently still focused on the diagnostic motif based on commercial contrast agents and additional functional imaging modules such as diffusion-weighted imaging. This model has been applied comprehensively and thoroughly to patient screening, tumor staging, pathological characterization, surgical planning, treatment decision, response evaluation, liver/peritoneal metastasis detection, neoplasms/implants, metastases/benign focal liver lesions distinction, and surveillance of CRC and its relapses within fibrotic changes^145,146^. The application of MNP-based multifunctional platforms in the theranostics of CRC emphasizes the preclinical stage due to hybrid system-associated multiple performance matching, complex evaluation processing, and potential biocompatibility considerations. Nevertheless, some prescient research achievements have predicted the successful clinical transformation of magnetic nanocomposites.

With the first clinical realization of online MRI-mediated adaptive tumor radiotherapy^147^, this approach overcame the limitations of frequently-used CT-based radiotherapy such as poor soft tissue definition and unfriendly administration, to result in considerable clinical benefits regarding MRI-guided daily adjustment of treatment regimens^148^. Furthermore, hybridization of MRI contrast agents and radiotherapy sensitizers is a treatment strategy that will soon move into clinical trials. Comprehensive consideration of sensitive diagnosis, effective treatment, tissue penetration and biocompatibility, and iron oxide (IO) nanocrystals with MRI and NIR photothermal effects are potential candidates for clinical transformation. In a preclinical study, monodisperse IO NPs with a diameter of 150 nm were synthesized by ligand-assisted co-precipitation and optimized by adjusting the amount of ligand. The NIR-IO NPs exhibited high photothermal conversion efficiency (21.2%) and T2-weighted MRI capability. They were not effective against CRC cells without irradiation, whereas significant cell killing and apoptosis induced by the changes of protein secondary structure and membrane permeability were demonstrated under 808 nm laser irradiation. Magnetic field-enhanced tumor accumulation greatly improves the T2-weighted MR signal (3-fold higher than a commercial MR contrast agent, Resovist^®^) and photothermal efficacy (~53 °C) for cancer treatment. The novel NIR-IO nanocrystals have great potential for MRI-coordinated targeted therapy of CRC^149^.

In brief, MRI-associated diagnostic and therapeutic protocols have been or are being developed with promising clinical value, and will eventually provide the theoretical and technical foundation for the accurate diagnosis and effective treatment of CRC.

## Concluding remarks and future perspectives

In this comprehensive review, recent advances in MNP-based multifunctional platforms for MRI-guided multimodal diagnosis and treatment of CRC are discussed. Continuously clarified carcinogenesis combined with universal preparation methods and modification strategies allow more rational and effective theranostic applications of MNPs in CRC treatments. The excellent physicochemical and biological properties of MNPs have shown that the next generation of molecular probes will focus on MRI-directed, MHT-enhanced cancer therapy and MNP-integrated multimode imaging, especially the PET/MRI diagnostic modality. The combined system integrates the complementary strengths of these two approaches (high sensitive anatomic resolution of MRI and excellent functional imaging of PET) into a single examination with reduced ionizing radiation, thereby increasing patient convenience and reducing medical costs. With the introduction of a clinical PET/MRI system, the hybrid pattern offers multiple diagnostic benefits to CRC patients, such as identifying the characteristics of liver lesions by introducing a hepatocyte-specific MRI contrast agent (gadoxetic acid), excluding extrahepatic disease, detecting additional liver metastases, and evaluating the treatment efficiency with multiple parameters, all of which cannot be achieved using a single PET, MRI, or conventional PET/CT combination^150^.

Despite encouraging achievements, some concepts remain theoretical, and certain internal logical mechanisms are vague. To achieve the clinical application of MNP-based theranostics, intensive efforts should be devoted to overcome the following challenges.

1. Mass preparation of monodispersed and reproductive MNPs. The *in vivo* biological effects of MNPs depend on their physicochemical characteristics (size, morphology, composition, and surface chemistry), which are vital and are determined by the synthetic process. Several strategies, such as separating the nucleation and growth stage, optimizing process parameters, and assisting the process with a polymer or surfactant, are under investigation to obtain high-performance MNPs^151^.
2. The nanotoxicity of MNPs. The off-target toxicity of MNPs is derived from the Fenton reaction of Fe particles to produce reactive oxygen species in biological systems and oxidation reactions of magnetite to form maghemite^152^. Surface coating with polymers, organic surfactants, and inorganic metals are effective ways to mask the oxidative sites, passivate MNPs, and reduce toxicity and the risk of DNA damage^153^.
3. Exploration and translation of new clinically relevant biomarkers. Cross-talk between signaling pathways and the emergence of resistance targets are reasons leading to drug failure. Thorough investigation and characterization of CRC-associated mutations will provide valuable information to treat CRC patients to enhance their diagnostic, prognostic, and therapeutic responses^154^.
4. Standardization of the methodology to study the long-term effects of MNPs on CRC biological systems. Similar to silver NPs, the exposure duration of cancer cells to MNPs determines the difference in biological effects. Therefore, the development of standardized methods to define the long-term interaction between magnetic DDS and the living body is urgent^155^.

Once these obstacles are overcome, multifunctional MNP-based DDS should show excellent capabilities in the accurate diagnosis and personalized therapy of malignant tumors.

## Figures and Tables

**Table 1:** Description of chemical preparation methods of MNPs and their strength and weaknesses

Method	Procedure	Strength	Weakness	Reference
Co-precipitation	The precipitation of metal salts under alkaline conditions to produce MNPs at room temperature or elevated temperature	Facile, convenient Cost-effective Easy to implement Less hazardous reagents Large-scale preparation	Unstable Polydisperse	^71^
Thermal decomposition	The decomposition of organometallic compounds and oxidation in high boiling point organic solvents containing stabilizing surfactants	Highly monodisperse Controlled structure and performance	High temperature Complicated MNPs dissolved in non-polar solvents	^72^
Hydrothermal	A phase transfer and separation process at the interfaces of the liquid, solid, and solution phases at high temperature (130–250 °C) and high pressure (0.3–4 MPa)	Simple Low cost Water dispersible Controlled morphology High purity and crystallinity	High temperature High pressure	^73^
Microemulsion	MNPs are generated by mixing inorganic salt and precipitating agent contained in the oil/water or water/oil nanodroplets	Adequate Versatile Controlled size and shape	Low yield Complicated purifying procedure	^75^
Sol-gel	Hydrolysis and polycondensation of metal precursors, metal, or metalloid element surrounded by various reactive ligands to form a “sol,” then dried by solvent removal or chemical reaction to form “gel,” followed by heat treatment for MNP harvesting	Pure Stoichiometric Monodisperse Large size Controlled structure	Low stability in aqueous solution	^76,77^
Polyol synthesis	It is based on a transfer and separation mechanism occurring at the interfaces of the metal precursor (solid), organic solvent (liquid) and water solution containing polyol derivatives.	Simple, reproducible Monodisperse Controlled morphology Cost effective Good crystallinity Excellent magnetic property	High temperature High pressure Toxic organic solvents	^78^

MNPs, magnetic nanoparticles.

**Table 2:** Polymer-based encapsulation techniques of MNPs

Methods	Procedure	Description	Reference
Nanoprecipitation	Dropwise addition of organic solution containing preformed polymer and MNPs into an aqueous phase with or without surfactant, under moderate agitation, the nanocapsules are instantaneously formed on the interface of both phases	Two phases are miscible Organic solvents are highly volatile Organic phase can be a mixed solvent Aqueous phase can be a mixed non-solvent	^81^
SEE	It consists of simple emulsion formation, solvent evaporation, polymer precipitation, and particles formation	There are oil/water and water/oil methods Organic phase should be non-miscible but can dissolve polymers Particle characteristics are controlled by adjusting procedure parameters, such as organic/water ratio, surfactant, stirring rate, polymer amount, and evaporation rate	^82^
DEE	Primary emulsion: dispersion of an aqueous phase containing MNPs in a non-miscible organic solvent under ultrasound and surfactant Second emulsion: the primary dispersion is added to a second solution containing the stabilizing agent under sonication Nanocapsules formation: NPs are obtained after evaporation of the solvents	It is classified as W/O/W or O/W/O emulsion It is suitable for the co-encapsulation of both hydrophilic and hydrophobic drugs and/or MNPs	^83^
LBL	LBL is a stepwise adsorption and assembly process based on spontaneous electrostatic attraction between oppositely charged components at supersaturating polyelectrolyte concentration, which leads to the adsorption of polyelectrolyte onto an oppositely charged particles surface	It is possible to control the size, shape, and thickness of multilayer nanocapsules The polymer should have sufficiently charged groups to provide stable adsorption on the oppositely charged surface Besides electrostatic interaction, hydrogen bonding and covalent bonding are also drivers for multilayer nanocapsule preparation	^84^

NPs, nanoparticles; MNPs, magnetic nanoparticles; W/O/W, water-oil-water; O/W/O, oil-water-oil.

**Table 3:** The theranostic applications of MNPs-based DDS in colorectal cancer treatment

NanoDDS	Preparation method	Tumor model	Diagnostic mode	Therapeutic mode	*In vivo* efficacy	Reference
PTX-SPIO-PEALCa micelle	Polymer self-assembly	LoVo xenograft tumor	MRI	Chemotherapy	The tumor volumes of PTX-SPIO-PEALCa, Taxol, and PBS groups at day 30 were 65.0 ± 8.4, 598.7 ± 77.4, and 1050.7 ± 54.4 mm^3^, respectively	^112^
PTX-PFOB-(PLGA-PEG)	Emulsion evaporation	CT-26 xenograft tumor	MRI	Chemotherapy	Two-fold reduction of tumor growth compared to control and Taxol^®^ groups	^113^
5-FU-magnetite-PLGA	O/W/O/W multiple emulsion and solvent evaporation	CT-26 allograft model	MRI	Chemotherapy	The nanocapsules showed more efficient tumor volume inhibition (100 mm^3^ at day 21) than 5-FU alone (1500 mm^3^)	^114^
Cisplatin-magnetite-P(MAA-g-EGMA)	Hydrolytic alkaline precipitation	HT-29 xenograft tumor	MRI PET CT	Chemotherapy	The tumor volumes of nano-assemblies+external magnetic field, nano-assemblies, and free cisplatin at day 38 were 300, 400, and 900 mm^3^, respectively	^115^
Oxaliplatin-SPIO-MWNTs-PEG	Polyol process	HCT-116 tumor-bearing mice	MRI	Chemotherapy	Nanotheranostics showed more effective tumor inhibition than oxaliplatin, with trivial weight loss (6.25%) and organ toxicity	^116^
PTX-F127-SIONR	Hydrothermal method	CT-26 xenograft tumor	MRI	Chemotherapy	PTX-F127-SIONR exhibited higher therapeutic response and lower tumor growth than PTX group, which showed comparable tumor size to control group, and 100% death at day 34	^117^
DOX-Fe_3_O_4_ MNPs	Co-precipitation	HT-29 cells in vitro	MRI	Chemotherapy	DOX-MNPs showed higher cytotoxicity (IC50 = 0.245 μmol/L) than free DOX (IC_50_ = 0.757 μmol/L)	^118^
MANPs-PTX	One step oxidation method	CT-26 xenograft tumor	MRI	Chemoradiation therapy	MANPs-PTX inhibited tumor growth of 96.57%; other treatments showed insufficient efficacy	^119^
Anti-MG1-HNPs	High temperature hydrolysis reaction	CC-531-implanted Wistar rats	MRI	PTA	The tumor inhibition rates of anti-MG1-HNPs, HNPs, and control groups were 38% ± 29%, 14% ± 17%, and 7% ± 8%, respectively	^120^
Aptamer-Au@SPIONs	Microemulsion	HT-29 cells in vitro	MRI	PTT	80% cell inhibition rate, at 500 µg/mL system concentration and 820 nm NIR exposure	^121^
Au-HNPs-scFv	–	SW1222 xenograft tumor	MRI	PTT	The tumor volumes of HNPs-scFv, laser only, non-targeted HNPs, and control groups were 72 ± 7, 161 ± 15, 195 ± 10, and 193 ± 18 mm^3^, respectively	^122^
A33scFv-HNPs	Thermal decomposition	SW1222 cellsHT-29 cells in vitro	MRI	PTT	After 6 min treatment of 808 nm laser, > 53% of SW1222 cells were involved with apoptosis-related cell death while < 5% occurred in HT-29 cells	^123^
Au@Gd_2_O_3_:Ln (Ln = Yb/Er)	Seed-mediated growth method	CC-531 xenograft tumor	MRI	PTT	Under 808 nm NIR light irradiation for 5 min, the tumor temperature increased by ~19.5 °C in the NPs group, showing a stronger PTT effect than the control (~7.5 °C)	^124^
HANs	Thermal decomposition	HT-29 xenograft tumor	MRI PAI	Chemotherapy PTT	The tumor volume of the HANs+laser group remained ~0 without a relapse, which was reduced slightly in the HANs alone group, while the tumor grew rapidly after laser treatment without the HANs and PBS groups	^125^
MGO-PEG-CET	Co-precipitation	CT-26 BALB/c mice	MRI	Chemotherapy PTT	The relative tumor volumes of control, DOX, MGO-PEG-CET/DOX, MGO-PEG-CET/DOX+magnet, and MGO-PEG-CET/DOX+magnet+laser were 12.1, 10.1, 9.5, 5.8, and 0.42, respectively	^126^
Fe-CPNDs	Coordination reaction	SW620 xenograft tumor	MRI	PTT	Complete tumor ablation at day 20	^127^
Hypericin-SPIONs	Co-precipitation	HT-29 cells in vitro	MRI	PDT	Cell proliferation was completely abolished at 2 µmol/L of hypericin and 60 h of illumination time	^128^
SiRNA plasmid-Au	–	LoVo bearing nude mice	MRI	Gene therapy	Bag-1 protein level was silenced to 60% of control, and caused the debasement of Wnt pathway	^129^
SN-38/USPIO-siRNA-PEG	O/W emulsion and solvent evaporation	LS174T bearing nude mice	MRI	Chemotherapy Gene therapy	The tumor volumes of SN-38/USPIO/siRNA, SN-38/USPIO, and SN-38 groups were 340 ± 52, 591 ± 125, and 1,150 ± 362 mm^3^, respectively	^130^

SPIO, superparamagnetic iron oxide; SIONR, SPIO nanorods; USPIO, ultra-small SPIO; Fe_3_O_4_, iron-oxide; PFOB, perfluorooctyl bromide; MWNTs, multiwalled carbon nanotubes; MNPs, magnetic nanoparticles; MnO_2_, manganese dioxide; HNPs, hybrid magnetic gold nanoparticles; HANs, hybrid anisotropic nanoparticles; CPNDs, coordination polymer nanodots; Au, gold; Gd, gadolinium; Ln, lanthanide; Yb, ytterbium; Er, erbium; MGO, magnetic graphene oxide; PEALCa, PEG-P(Asp-DIP)-P(Lys-Ca); PEG, polyethylene glycol; P(Asp-DIP), poly(N-(N′,N′-diisopropylaminoethyl) aspartamide); P(Lys-Ca), poly (lysine-cholic acid); PLGA, poly(lactide-co-glycolide); P(MAA-g-EGMA), poly(methacrylic acid)-g-poly(ethyleneglycol methacrylate); F127, pluronic F127; MANPs, MnO_2_ functioned albumin nanoparticles (ANPs); PTX, paclitaxel; 5-FU, 5-fluorouracil; DOX, doxorubicin; SN-38, 7-ethyl-10-hydroxycamptothecin; PBS, phosphate-buffered saline; O, oil; W, water; IC_50_, half maximal inhibitory concentration; MG1 mAbs, a monoclonal antibody localized to rat colorectal liver metastasis cells; scFv, single-chain variable fragment; A33, an antigen presented on some CRC cells such as SW1222; CET, cetuximab, an EGFR monoclonal antibody; siRNA, small interfering RNA; Bag-1, Bcl-2-associated athanogene 1; MRI, magnetic resonance imaging; PET, positron emission tomography; CT, computed tomography; PAI, photoacoustic imaging; PTA, photothermal ablation; PTT, photothermal therapy; PDT, photodynamic therapy; NIR, near infrared; CRC, colorectal cancer, LoVo cells, human CRC cells; CT-26 cells, murine colon cancer cells; HT-29 cells, human colon cancer cells; HCT-116 cells, human colon cancer cells; CC-531 cells, rat colorectal liver metastasis cells; SW1222 cells, human CRC cells; SW620 cells, human CRC cells; LS174T cells, human colon cancer cells; DDS.
